# Feasibility of Hot Melt Extrusion in Converting Water-Based Nanosuspensions into Solid Dosage Forms

**DOI:** 10.3390/pharmaceutics17050662

**Published:** 2025-05-17

**Authors:** Erasmo Ragucci, Marco Uboldi, Adam Sobczuk, Giorgio Facchetti, Alice Melocchi, Mauro Serratoni, Lucia Zema

**Affiliations:** 1 PhormulaMi Research Group, Sezione di Tecnologia e Legislazione Farmaceutiche “Maria Edvige Sangalli”, Dipartimento di Scienze Farmaceutiche, Università degli Studi di Milano, Via Giuseppe Colombo 71, 20133 Milano, Italy; erasmo.ragucci@unimi.it (E.R.); marco.uboldi@unimi.it (M.U.); lucia.zema@unimi.it (L.Z.); 2 Novartis Pharma AG, 4056 Basel, Switzerland; adam.sobczuk@novartis.com (A.S.); mauro.serratoni@novartis.com (M.S.); 3 Dipartimento di Scienze Farmaceutiche, Università degli Studi di Milano, Via Venezian 21, 20133 Milano, Italy; giorgio.facchetti@unimi.it

**Keywords:** hot melt extrusion, nanosuspension, granules, immediate release, modified release

## Abstract

**Aim:** In addition to numerous benefits provided by nanosuspensions (NSs) (e.g., enhanced saturation solubility, increased area for interaction with fluids), they suffer from major stability, handling and compliance issues. To overcome these challenges, we evaluated the feasibility of hot melt extrusion (HME) in transforming a cinnarizine-based NS, selected as a case study, into granules for oral intake. **Methods:** Thermoplastic polymers, in principle compatible with the thermal behavior of the selected drug and characterized by different interaction mechanisms with aqueous fluids, were used as carriers to absorb the NS and were processed by HME. **Results**: The extruded granules pointed out good physio-technological characteristics, a drug content > 85% with coefficient of variation (CV) < 5% and tunable in vitro performance coherent with the polymeric carriers they were composed of. Particle size as well as the solid state of cinnarizine was checked using several analytical techniques in combination (e.g., DSC, SEM, FT-IR, Raman). Depending on the composition of the granules, and specifically for formulations processed below 85 °C, the drug was found to remain crystalline and in the desired nanoscale. **Conclusions:** HME turned out to be a versatile process to transform, in a single-step, NSs into multi-particulate solid products for oral administration showing a variety of release profiles.

## 1. Introduction

In recent years, the advent of digital technologies, such as artificial intelligence, machine learning and high-throughput screening methods, has led to the development of numerous new chemical entities (NCEs) having a high affinity for the biological target and enhanced pharmacological activity [1,2,3]. In this field, major efforts were devoted to the development of solid products for oral administration, like tablets and capsules, containing such NCEs, to ensure high patient compliance and low manufacturing costs, thus facilitating their spread in the pharmaceutical market [4,5].

However, due to their relatively large molecular weight as well as chemical structures, many NCEs present permeability issues through the gastrointestinal (GI) membranes and poor solubility in the GI fluids, which could affect absorption and bioavailability upon oral intake [6,7].

To counteract solubility constraints, nano-sizing poorly soluble drugs, resulting in the attainment of NSs, has been widely investigated [8,9,10].

NSs are submicron colloidal, heterogeneous, aqueous dispersions of practically insoluble active ingredients with a particle size in the 1–1000 nm range, which are stabilized in their crystalline state by different excipients, such as surfactants and thickening agents [11]. They can be attained through top-down or bottom-up methods, the former relying on the application of mechanical stress, while the latter on a controlled condensation/aggregation of particles from a molecular dispersion [12]. Overall, NSs are characterized by many advantages such as (i) enhanced specific surface area of the drug bulk and improved saturation solubility [13], which would result in a faster dissolution rate according to the Noyes–Whitney dissolution model [14], (ii) greater surface of interaction of drug particles with the solvent [15] and (iii) enhanced mucoadhesion, increasing their GI residence and thus the time available to complete the dissolution process [1]. However, they are ale characterized by physical and chemical stability issues, resulting, for instance, in sedimentation, aggregation, solid-state transformation, dimensional alteration and crystal growth [10,16,17].

To reduce these phenomena, removing water and transforming NSs into more convenient solid products, without losing the advantages related to the presence of the drug at the nano-scale, would be fundamental. As a first stage, researchers tested well-established spray-drying and freeze-drying technologies on NSs, improving their stability and handling [18,19,20,21,22,23]. However, such traditional drying methods suffer from high energy consumption and long processing times, representing only the preliminary step in the manufacturing of a solid dosage form. On the other hand, attaining the final medicinal products taking advantage of the so-called continuous manufacturing approach has recently raised a lot of interest [24,25,26,27,28]. Indeed, it was demonstrated a suitable way to increase manufacturing efficiency as well as production rate while reducing expenses per unit, especially if coupled with real-time quality controls. In this respect, hot melt extrusion (HME) using Soluplus™ as the polymeric carrier was tested as an alternative and single-step process to transform NSs into solid products and was named nano-extrusion [29,30,31]. Indeed, hot-processing and particularly HME has recently emerged as a promising and versatile technology [31,32,33,34,35,36], not only towards the manufacturing of solid dispersions, but also to attain a variety of delivery systems (e.g., granules, capsules, pellets, tablets, rings, implants, inserts), ranging from immediate to modified release, and intended for diverse administration routes.

Based on the above-mentioned considerations, in this work we intended to widen the range of formulations undergoing HME to directly transform a water-based NS into solid dosage forms for oral intake. More in detail, HME was carried out to manufacture, in a one-step process, multi-particulate systems (i.e., granules) starting from a cinnarizine-containing NS (CN NS), which was employed as a noteworthy case study. Indeed, CN not only has a challenge of thermal behavior (i.e., relatively low melting temperature, T_m_) but could also represent a model compound for the class II of the Biopharmaceutical Classification Systems (BCSs), in which many NCEs fall [37]. For the sake of versatility, polymeric formulations in a diverse range of release performances (i.e., immediate and prolonged release, targeted release to specific sites of the GI tract) were evaluated.

## 2. Materials and Methods

### 2.1. Materials

In this study, we used polyethylene oxides (PEOs) with different molecular weights (Sentry POLYOX^TM^ WSR N10 LEO NF, PEO N10; Sentry POLYOX^TM^ 303 LEO NF, PEO 303; Iff, Milan, Italy); polymethacrylates (Eudragit^®^ RL PO, Eu RL; Eudragit^®^ RS PO, Eu RS; Eudragit^®^ E PO, Eu E; Evonik, Essen, Germany); glycerol (GLY; A.C.E.F., Milan, Italy); polyethylene glycol 8000 (PEG; Clariant Masterbatches, Milan, Italy); triethyl citrate (TEC; Sigma Aldrich, Milan, Italy); sodium starch glycolate (EXPLOTAB^®^ CLV, EXP; JRS PHARMA, Rosenber, Germany); hydrated dextrates (EMDEX^®^, EMD; JRS PHARMA, Rosenber, Germany); glycerol (GLY; Pharmagel, Lodi, Italy); hydroxypropyl methylcellulose (Pharmacoat^®^ 603; Shin-Etsu Chemical Co., Tokyo, Japan); copovidone (Kollidon^®^ VA 64, BASF SE, Ludwigshafen, Germany); dioctyl sulfosuccinate sodium salt (Sigma Aldrich, Darmstadt, Germany); sodium lauryl sulphate (Carl Roth, Karlsruhe, Germay); potassium dihydrogen phosphate (KH_2_PO_4_) (VWR, Milan, Italy); potassium monohydrogen phosphate (K_2_HPO_4_) (VWR, Milan, Italy); methanol (VWR, Milan, Italy); HCl (VWR, Milan, Italy); sodium cloride (NaCl) (VWR, Milan, Italy); epoxy resin (Araldite^®^, Velcro Brand, Deinze, Belgium).

CN NS was kindly supplied by Novartis Pharma AG (Basel, Switzerland) and contained d-a-tocopheryl polyethylene glycol 1000 succinate (TPGS, Sigma-Aldrich, Basel, Switzerland) as a steric stabilizer [38]. CN is a highly lipophilic BCS Class II drug, having a highly pH dependent solubility (0.29 mg/mL at pH 2, 0.017 mg/mL at pH 5, and 0.002 mg/mL at pH > 6.5) and T_m_ of 118–122 °C [39,40]. CN NS was manufactured by Novartis Pharma AG, according to previous studies [32].

### 2.2. Methods

#### 2.2.1. Preparation of the CN NS

CN NS was attained via wet media milling (WMM), starting from a stabilized raw aqueous suspension (4:35:1, drug:water:stabilizer in *w*/*w*). Initially, stabilizers screening was carried out by Novartis Pharma AG (Basel, Switzerland). TPGS, hydroxypropyl methylcellulose and Kollidon^®^ VA 64 alone or in combination with anionic surfactants (i.e., dioctyl sulfosuccinate sodium salt and sodium lauryl sulphate) were considered, testing the resulting NS for key quality parameters (e.g., particle size distribution, viscosity and microscopically), as before described [17,41]. From this pre-formulation study, the TPGS-containing NS resulted in the most promising candidate. With respect to the preparation of the stabilized raw aqueous suspension, 2.5% *w*/*w* TPGS was first dissolved in bidistilled water. Then, micronized CN powder (10% *w*/*w*) was dispersed within the stabilizer solution (magnetic stirring for about 12 h), until a homogeneous suspension was obtained. The latter underwent WMM using an agitator bead mill (DELTAVITA^®^ 15-300, Netzsch, Selb, Germany) equipped with 600 mL silicon carbide milling chamber and yttria zirconia beads of 0.1 mm in diameter (mass = 1547 g; ρ_bulk_ = 3.58 g/cm^3^, Netzsch, Selb, Germany). The chamber was filled up to 80%. The pumping rotation speed was set at 30–80 rpm (13–33 L/h), while the mill rotation speed was 10 m/s. The temperature of the suspension was kept constant at the inlet (13 °C) and at the outlet (18 °C) of the milling chamber and the total processing time was around 6 h. The total mass of the CN NS manufactured via different batches was 2.5 kg (each batch size = 1 kg).

#### 2.2.2. Particle Size (Z-Average) and Polydispersity Index (PdI) via Dynamic Light Scattering (DLS)

Z-average and PdI of CN nanoparticles (n = 3) were measured using Zetasizer nano ZSP (Malvern Panalytical Ltd., Milan, Italy). Before the analysis, samples were diluted (1:100) using a 0.1 mM NaCl solution and equilibrated for 120 s to ensure proper conditioning. The temperature was set at 25 °C and each analysis involved 10 measurements (100 s duration each).

The stability of the CN NS, stored in a refrigerator (VWR, Basel, Switzerland; 3 ± 1 °C), was checked measuring the Z-average and PdI at pre-established time periods (i.e., up to 18 months).

#### 2.2.3. Drug Content

CN content (n = 3) was determined using high-performance liquid chromatography (HPLC; HP 1100 ChemStations, Agilent Technologies, Milan, Italy). The latter was equipped with an UV-Vis detector and a reverse-phase column L-column (InertClone ODS, 150 × 4.6 mm 5 μm ODS(3) 100 Å, Phenomenex Inc., Aschaffenburg, Germany), maintained at 30 °C. The mobile phase, i.e., mixture of methanol and pH 3.5 phosphate buffer [42] in water for HPLC (70:30, *v*/*v*), was filtered before use (0.45 µm membrane filter) and pumped at a flow rate of 1 mL/min at 30 °C. The time of the run was set at 10 min. The sample to be evaluated (25 mg) was initially dispersed in 15 mL of mobile phase and then ultrasonicated at 25 °C for 30 min to ensure complete CN dissolution. The resulting liquid was filtered through a 0.22 μm syringe filter and transferred into a HPLC vial kept at 30 °C, from which 10 μL were automatically injected into the HPLC system for being assayed spectrophotometrically (retention time = 4.7 min, λ = 250 nm). The CN content was calculated using a purposely built calibration curve in the 0.25–500 µg/mL range (y = 33.581x + 5.0951, R^2^ = 0.9998).

#### 2.2.4. Differential Scanning Calorimetry (DSC)

Thermal analysis was carried out by DSC (DSC Discovery, TA Instruments, Milan, Italy; DSC Stare System 1, Mettler-Toledo, Milan, Italy). Samples were accurately weighed (3–7 mg) and tested in sealed and pierced aluminium pans. The analysis was performed under a nitrogen atmosphere (N_2_ 80 mL/min), setting a single heating ramp (25 °C to 200 °C, rate 10 °C/min). To test the CN NS, the latter was oven dried (40 °C, 8 h; VWR, Milan, Italy) before performing the analysis.

#### 2.2.5. HME

HME was performed in a twin-screw extruder (Haake™ MiniLab II, Thermo Fisher Scientific, Milan, Italy), equipped with counter-rotating conical screws and a rod-shaped die (custom-made in aluminum ø = 1.80 mm) [43,44]. The mixtures to be tested were prepared in a mortar and manually fed into the barrel of the equipment. The extrudability of placebo formulations was first assessed to identify suitable operating conditions, as described in Section 3. Specifically, the process was performed setting different excipient-wise temperatures along the barrel, which were selected based on literature findings and preliminary DSC experiments), while the screw speed was modified in real-time to avoid any increase of the torque above 200 N·cm. During manufacturing, extruded rods were manually pulled out of the die, discarding the first and the last parts attained. Then, they were manually cut into 1.8 × 2 mm granules, which were checked for weight (analytical balance BP211, Sartorius, Milan, Italy) height and diameter (digital caliper, Milan, Italy) before being stored in a static oven at 40 °C (VWR, Milan, Italy).

#### 2.2.6. Fourier-Transform Infrared (FT-IR) Spectroscopy

FT-IR analyses were carried out using an FT-IR Spectrometer (Spectrum One, Perkin Elmer, Rodgau, Germany), operating in the spectral region between 4000 and 650 cm^−1^. The data were analyzed by transmittance technique with 32 scansions and 4 cm^−1^ resolution.

#### 2.2.7. Confocal Raman Microscopy (CRM)

An Alpha 300 R confocal imaging system (WITec, Ulm, Germany) was used to collect both Raman spectra and relevant maps. The excitation wavelength of 532 nm, with a laser intensity of 12–15 mW, was provided by an Nd:YAG laser (WITec, Ulm, Germany). Offset correction was performed for each study by calibration against a silicate substrate. The hyperspectral grating was G1: 600 g/mm, BLZ 500.00 nm, with a spectral center at 2100 cm^−1^. Scans were performed with an integration time of 50–100 ms using the WITec TrueSurface; an Mk3 extension with real-time large area topographic imaging module microscope was equipped with a Zeiss LD Plan-Neofluar 63× NA 0.75 objective (Zeiss, Oberkochen, Germany). For each sample, three fragments were randomly selected, embedded in epoxy resin, and kept at least for 24 h at room temperature to allow the resin to harden. The resulting resin blocks were cut using an ultra-microtome (Leica EM UC6, Wetzlar, Germany) to obtain level block cross-sections. Initially, large-area scans were acquired at low resolution (lateral 10 μm) to gain an overview of the spatial distribution and localization of the extrudate components. Subsequently, focused high-resolution area scans were carried out at 400 × 300 μm^2^ with a lateral resolution of 1 μm, and at 100 × 100 μm^2^ with a lateral resolution of 0.5 μm. Hyperspectral data processing, including offset, cosmic ray removal, baseline correction, spectral filter application, average spectral extraction and spectral map deconvolution was performed using WITec Project SIX Plus 6.2 software. The corresponding intensity maps were calculated by the application of WITec TrueComponent Analysis and thresholding. Reference material spectra, including those of CN and polymeric carriers, were obtained by single spectral measurements whereby characteristic peaks for each species were defined for hyperspectral processing.

#### 2.2.8. Scanning Electron Microscopy (SEM)

Following surface chemical modification by gold plasma evaporation (~8 nm), SEM images were acquired (SEM GEMINI 300, Carl Zeiss AG, Jena, Germany) using an accelerating voltage of 4.0 kV and setting ~6 mm as the working distance. For the CN NS, 5 μL was filtered (0.2 μm Nucleopore Track Etch membrane, Sigma Aldrich, Schnelldorf, Germany) and washed with 50 μL of ultrapure water before chemical modification.

#### 2.2.9. In Vitro Performance

In vitro performance (n = 3) was evaluated in an acidic buffer (pH 1.2) using a USP Apparatus 4 (Sotax, Allschwil, Switzerland) equipped with cells for powder/granules, in which samples of 25 ± 2 mg were inserted before starting the test. The apparatus was operated in an open configuration, i.e., having a continuous flow of fresh media into the cells (8 mL/min) and automatic sampling of the latter at pre-established time points (5 mL every 5 min). The fluids collected were then analyzed via HPLC following the procedure described in Section 2.2.3. Data were expressed as percentage of drug dissolved with respect to the actual drug content.

## 3. Results and Discussion

### 3.1. Selection of Polymeric Carriers

The main target of the present work was to demonstrate the possibility of using the HME technique to transform, in a single step, a model NS into solid units. These should maintain the initial advantageous characteristic of the NS (i.e., the selected drug in the crystalline state, with the desired nano-scale particle size distribution), while being able to provide a range of release patterns. In this respect, the first part of the investigation was focused on the selection of carriers suitable for HME and with a potential towards both immediate and modified release. Overall, extrudability of the formulations was deemed the pivot of the research, being the thermal behavior of the selected drug (and thus its stability at the processing temperatures involved) and that of the polymers both crucial for making the transformation feasible [33]. For this reason, T_m_, glass transition (T_g_) and degradation temperatures of all the materials in use were carefully considered [34]. In this respect, identifying suitable polymeric carriers was particularly challenging in view of the low CN T_m_. To avoid amorphization and to obtain a nanocrystal solid dispersion, HME temperatures should be kept to at least 20–30 °C below the drug melting point [35,36]. At the same time, proper processing generally requires operating at a minimum of 20 °C above the T_g_ of the polymeric carrier. For this reason, only polymers known to be extruded at temperatures ≤ 100 °C were included in the screening. Willing to attain final products characterized by both immediate (IR) and prolonged release (PR) profiles, polymers with different interaction mechanism with aqueous fluids were considered. In this respect, HME already demonstrated being a versatile technology, able to process a wide range of materials [32,45,46]. Based on the above-mentioned considerations, details on the polymeric carriers selected for the work are reported below:

- EXP: it shows a thermoplastic behavior under thermo-mechanical activation and in the presence of water and of another plasticizer, such as GLY, to avoid relevant retrogradation [47]. After either HME or injection molding, it still maintains the ability to disintegrate.
- PEO N10 and 303: non-ionic thermoplastic homopolymers of ethylene oxide having T_m_ < 75 °C. They are soluble in water, with swelling capacity dependent on the molecular weight. They could thus be used for the development of both IR and swellable/erodible PR-release systems [48,49,50].
- PEG: highly hydrophilic soluble polymer characterized by low T_m_ (around 60 °C), which is mainly used for the production of solid dispersions and IR systems [51];
- Eu RS, RL and E: thermoplastic polymethacrylates with different thermal and solubility characteristics [45,52]. More into detail, Eu E is a cationic copolymer soluble in acidic media (up to pH 5.0) with a T_g_ of about 48 °C. Eu RL and Eu RS are insoluble cationic polymers sharing the same molecular structure but differing in the content of ammonium functional groups. This results in diverse permeability properties, with T_g_ values around 70 and 67 °C, respectively.
- EMD: water-soluble and lactose-free materials composed of glucose monohydrate and different polysaccharides derived from starch, showing a hot-processing aptitude similar to the latter [26,43].

### 3.2. Blend Preparation and HME Trials

To assess the potential of HME for the intended target, the high amount of water of the NS represented a key aspect towards the hot-processability of the selected carriers. In this respect, the work was further challenged by the availability of a lab-scale equipment with a very short barrel. This represented a major constraint. Indeed, the operator was forced to incorporate the desired load of CN (and thus the associated NS volume) gradually into the polymeric carrier (i.e., by repeating the wetting, kneading and drying steps several times) before loading the formulation into the extruder. First trials were performed on samples composed of the sole polymer, eventually plasticized, to rule out the capability of the various formulations towards water (used as a mock-up of the CN NS) incorporation. The pre-test consisted of wetting the selected polymers with the same amount of water contained in a 100 mg/g NS aliquot. This way, it was possible to estimate the number of granulation drying (40 °C) steps needed to avoid the formation of a paste that cannot be fed into the extruder. Overall, complete loading of the liquid took from 1 to 6 repeated stages, depending on the polymeric carrier considered. After addition of the proper type and amount of plasticizers, which were selected based on literature data and on previous findings collected in the lab, formulations underwent HME. The operating conditions set are reported in Table 1. These were selected considering the aspect of the extruded rods at the die and to avoid any temperature increase over 100 °C, which might have caused CN amorphization.

Placebo extrudates showing either a non-uniform shape/diameter or characterized by extreme brittleness were discarded (i.e., EXP- and EMD-based ones). Then, drug-containing formulations were prepared by using either the CN micronized powder (i.e., reference extruded products manufactured for comparison purposes) or the CN NS, targeting a 10% *w*/*w* drug load in the final products. The granulation-drying steps and the operating conditions optimized for placebo formulations were employed.

**Figure 1:**
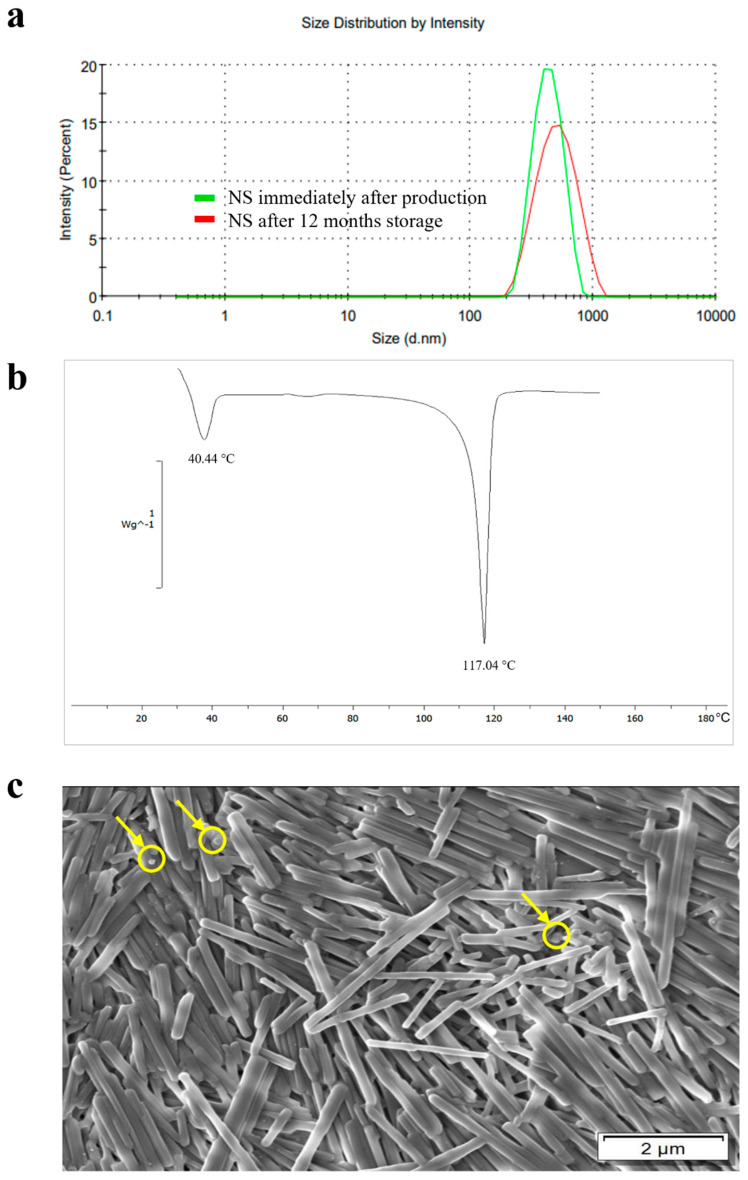
(**a**) Particle size and distribution of CN attained by DLS at different storage times together with the relevant (**b**) DSC thermogram and (**c**) SEM image showing the cylindrical and ellipsoidal-spherical (in yellow circles) geometries of CN nanoparticles.

The CN NS contained nanoparticles of ~500 nm with a PdI of 0.4 and was demonstrated dimensionally stable over time (Figure 1a). The thermal analysis of oven-dried aliquots of the NS highlighted the presence of two endothermic peaks, being the first related to the TPGS stabilizer (T_m_ = 40.44 °C) and the second sharp one ascribed to the CN melting (T_m_ = 117.04 °C) (Figure 1b) [53,54]. SEM was also performed to obtain a comprehensive morphological overview of the NS (Figure 1c) [55]. CN nanocrystals were characterized by two distinct shapes, i.e., cylindrical and ellipsoidal-spherical geometries, and resulted dimensionally polydisperse. Indeed, cylindrical needle-like particles, having a length between 0.4 and 4 μm, co-existed with rare spherical in-shape nanocrystals characterized by smaller dimensions (between 100 and 500 nm).

### 3.3. Granule Characterization

**Figure 2:**
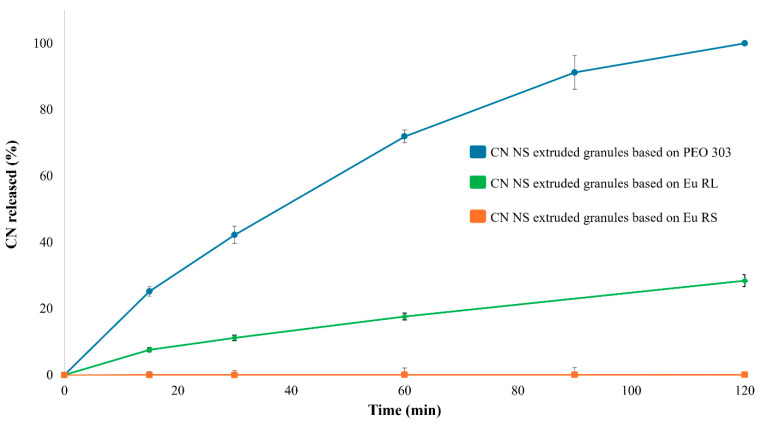
Release profiles (USP Apparatus 4 equipped with cells for powder/granules, acidic buffer pH 1.2 flowing at 8 mL/min, 5 mL of fluid sampled every 5 min) relevant to PR granules having different composition.

All the granules attained, both placebo and CN-containing ones, showed an analogous aspect and homogeneous dimensional characteristics. The drug load was always higher than 85% (CV < 5%), highlighting a good processability of the formulations investigated, intended as good dispersibility of the nanocrystals in the wet mass during the initial granulation step and then in the molten formulation undergoing HME. Micro-scale insights on the extruded products were then obtained taking advantage of Raman confocal microscopy. Furthermore, in vitro performance of the finished granules was consistent with the characteristics of the polymeric carriers they were composed of [52,56] and was confirmed after 12 months of storage. Focusing on PEO N10-, PEG-, and Eu E-based IR units, the whole amount of drug was dissolved within 30 min (data reported in the subsequent chapters). On the other hand, all the PR carriers investigated were able to slow down drug release, with 100%, 25%, and <1% of CN released after 2 h of testing when dealing with PEO 303, Eu RL and Eu RS, respectively (Figure 2). Ensuring proper control of the release rate of CN, which is a poorly soluble drug, from PR multi-particulate systems could be especially challenging when the drug is loaded into the system as a NS (i.e., a form intrinsically endowed with a higher solubility and dissolution rate in view of the particle size reduction up to the nanoscale). Indeed, in PR systems, the release rate must be controlled by the characteristics of the formulation (e.g., permeability through the inert matrix or through the polymeric network of the swelled one) and not unintentionally determined by the very low tendency to dissolve of the conveyed drug.

**Figure 3:**
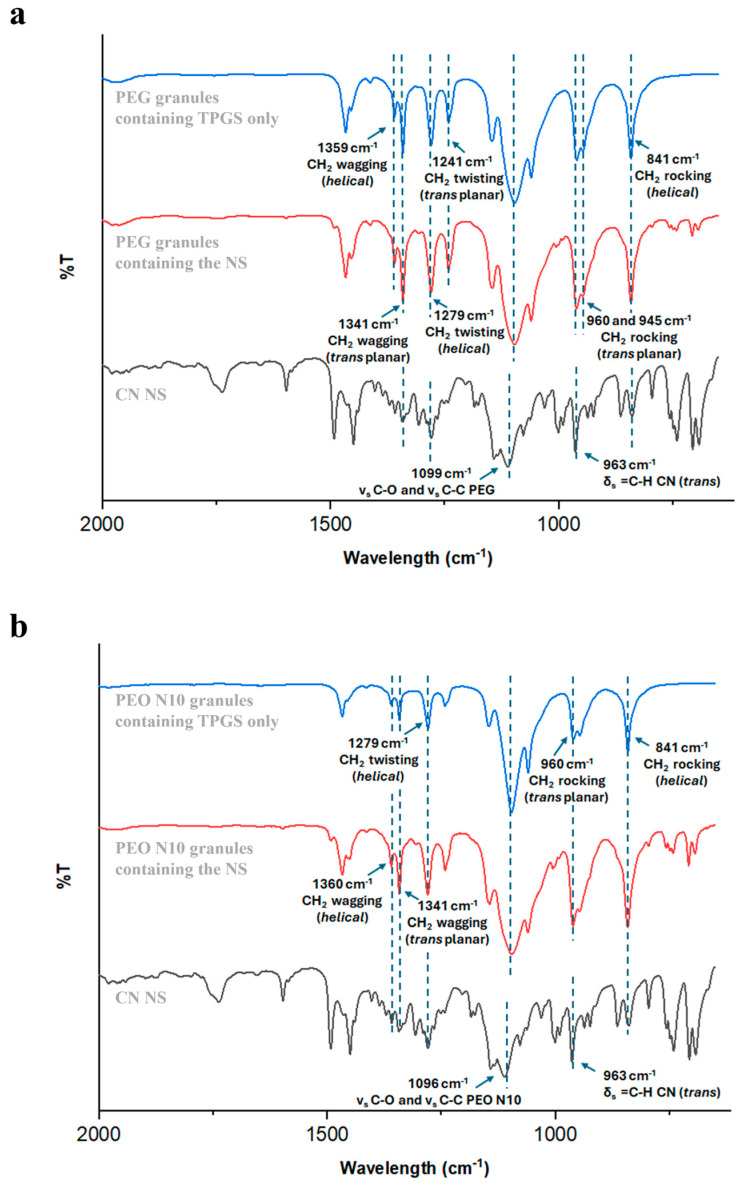
FT-IR spectra of (**a**) PEG- and (**b**) PEO N10-based granules.

Several analytical techniques were employed to assess whether the CN nanocrystals remained dispersed within the HME granules. More into detail, by determining diagnostic peaks relevant to different molecular entities included in the specimens, FT-IR analysis helped in preliminarily verifying the composition of the sample. The presence of CN was associated with an intense band at 963 cm^−1^, due to the =C─H bending within a double bond in trans configuration (Figure 3). In parallel, bands known for being characteristic of different polymer structures were ruled out. By way of example, the FT-IR spectra of formulations based on PEG and PEO N10 are reported in Figure 3 [57,58]. To complement the above-mentioned data, confocal Raman microscopy was selected as a suitable imaging approach to gain a deeper understanding of CN-polymeric carrier interactions and drug distribution within the polymer matrix. In particular, CN reference spectra were selected from the Novartis database, with spectral filters centred at 90, 119 and 218 cm^−1^ (lattice vibration frequencies), 1002 cm^−1^ (aromatic ring), 1597 cm^−1^ (aromatic ring) and 1654 cm^−1^ (C=C, double bond of the lateral chain) [59]. More specifically, the presence of characteristic shoulders in the diagnostic peaks at the lattice vibration frequencies were correlated to the presence of crystalline CN. On the other hand, based on previous literature findings [60], excipient spectral extraction was determined using filters centred at:

- 363 cm^−1^ (C-O-C bending vibration), 1481 cm^−1^ (CH₂ bending), 2846 cm^−1^ (symmetric stretching of CH₂ in a saturated bond) and 2887 cm^−1^ (OH stretching) for PEO N10, PEO 303 and PEG 8000 (oxyethylene group CH₂-CH₂-O-);
- 1450 cm^−1^ (CH₂ methylene group), 1726 cm^−1^ (C=O stretching of the carbonyl group of ester function) and 2948 cm^−1^ (symmetric and asymmetric stretching of C-H in methyl and methylene functions) for Eu polymers;
- 866 cm^−1^, 1739 cm^−1^ and 2940 cm^−1^ for TEC;
- 844 cm^−1^, 1751 cm^−1^ and 2886 cm^−1^ for TPGS.

DSC and SEM analyses were employed to gain a further confirmation on the FT-IR results, particularly regarding the possibility that the HME process might have caused CN amorphization or had an impact on morphology, size and distribution of the drug nanocrystals within the polymeric matrices. In this respect, illustrative results, i.e., those useful to deepen the peculiarities of the various case studies investigated, are briefly presented in the following paragraphs.

#### 3.3.1. PEG

**Figure 4:**
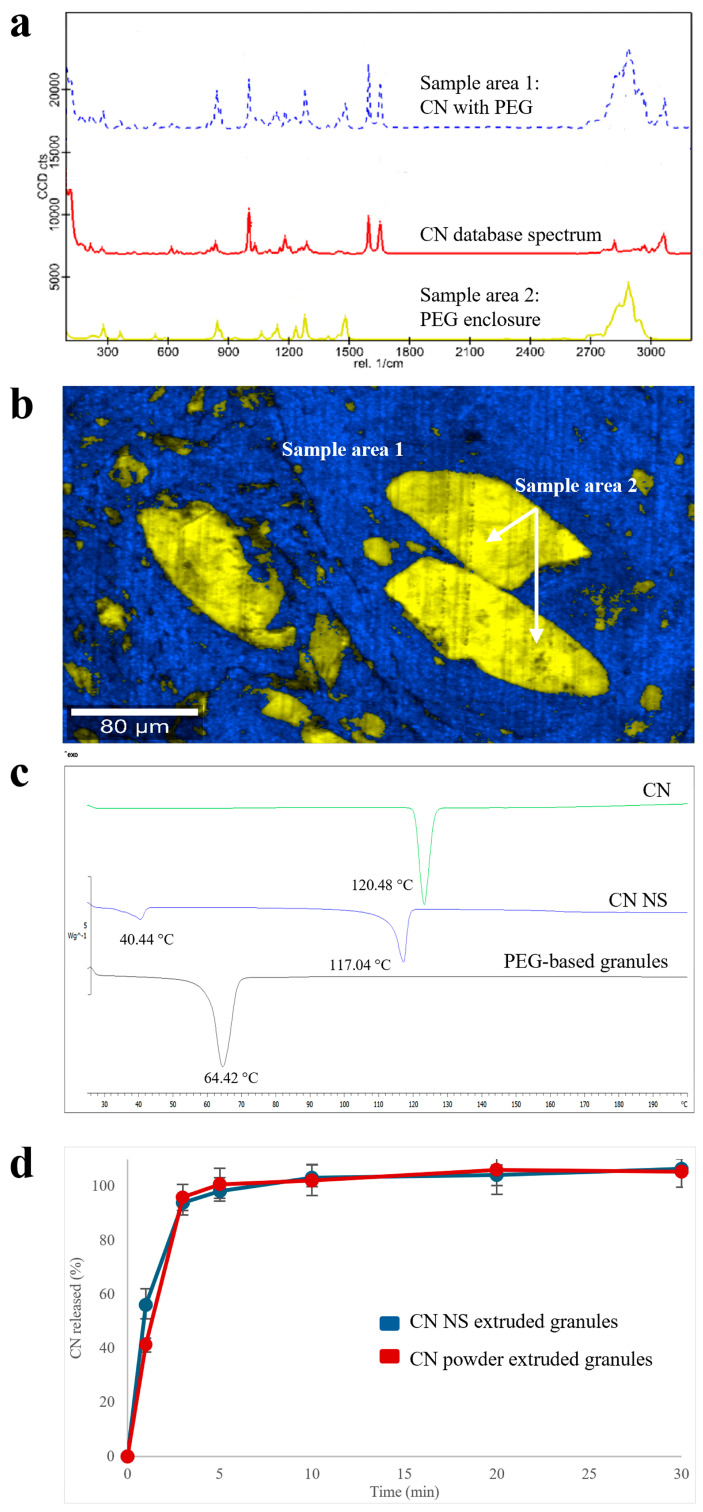
(**a**) Raman spectra, (**b**) Raman molecular map, (**c**) DSC thermograms and (**d**) release profiles (USP Apparatus 4 equipped with cells for powder/granules, acidic buffer pH 1.2 flowing at 8 mL/min, 5 mL of fluid sampled every 5 min) relevant to PEG-based products.

Raman spectra collected from different areas of PEG-based granules were useful in appreciating the spatial distribution of the two main molecular entities identified (Figure 4a): (i) an extruded homogeneous matrix, in which CN was mainly dispersed into the polymeric carrier (i.e., blue sample area 1 in Figure 4b), and (ii) well-demarcated PEG enclosures (i.e., yellow sample area 2 in Figure 4b). The presence of the latter was attributed to an inadequate melting of the polymer, probably as a consequence of the limited residence time of the formulation within the barrel of the lab-scale equipment available, coupled with a too low extrusion temperature.

Although the slight presence of the shoulders of diagnostic peaks at the lattice vibration frequencies in the CN spectra may inform about the presence of undefined quantities of crystalline CN, the DSC profile (Figure 4c) was only characterized by (i) in the presence of an endothermic sharp peak (T_m_ = 64.42 °C), which can be ascribed to the polymeric carrier melting point and (ii) in the absence of peculiar CN endothermic peaks, suggesting amorphization of the majority of CN in the granules. Analogous profiles were obtained with the reference extruded specimens containing CN powder as such, supporting the hypothesis of drug dissolution in the molten PEG. This result was in agreement with initial assessments of CN compatibility with different polymers carried out by DSC. Accordingly, the release performance of both extruded products (in acidic buffer, pH 1.2) was quite similar (Figure 4d). Only a slight difference could be noticed at the very beginning of the experiment. Indeed, after 3 min of testing, the amount of CN dissolved from the NS-based products was 15% greater than that relevant to samples containing the micronized drug powder.

#### 3.3.2. PEO N10

**Figure 5:**
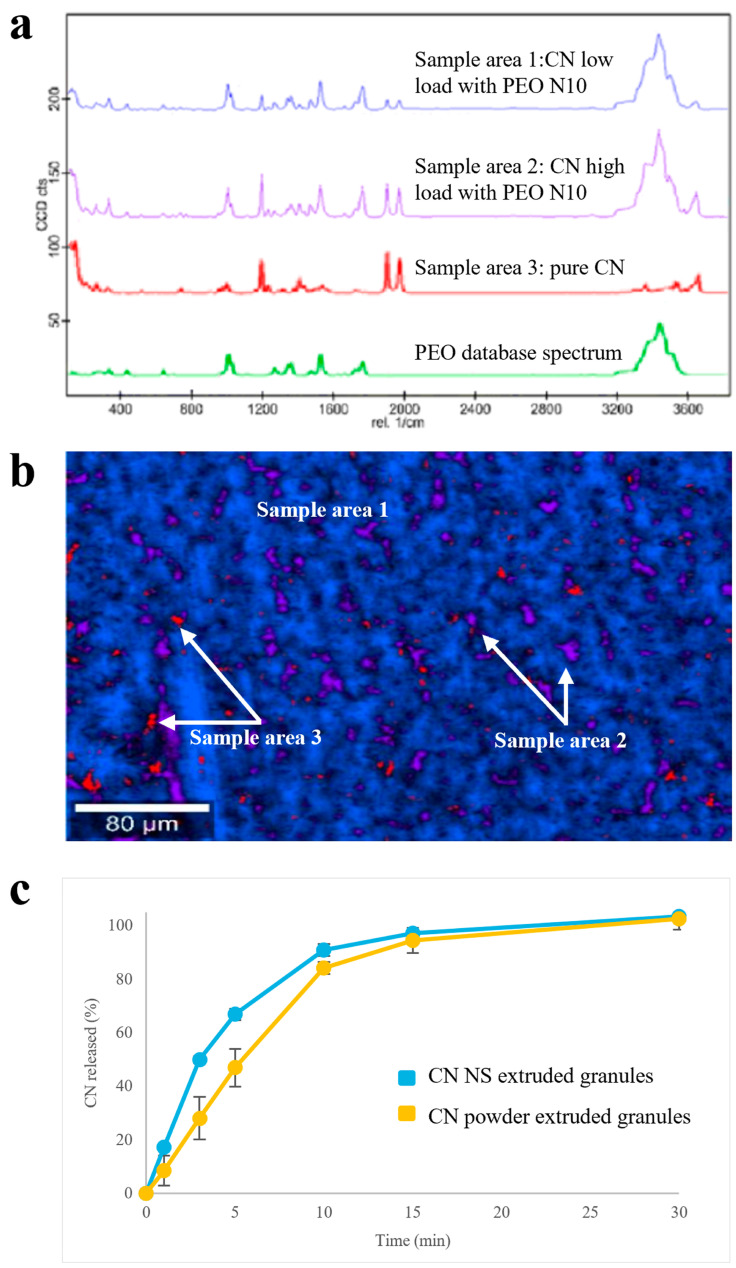
(**a**) Raman spectra, (**b**) Raman molecular map and (**c**) release profiles (USP Apparatus 4 equipped with cells for powder/granules, acidic buffer pH 1.2 flowing at 8 mL/min, 5 mL of fluid sampled every 5 min) relevant to PEO N10-based granules.

Focusing on Raman spectra of samples based on low-molecular weight PEO, areas with different composition could be identified (Figure 5a,b): a homogeneous polymeric matrix containing amorphous CN (i.e., blue sample area 1), in which spots of almost pure crystalline drug (i.e., red sample area 3) or of polymeric matrix very highly concentrated in CN (i.e., violet sample area 2) were randomly dispersed. The dissolution performance was coherent with the above-mentioned composition. Indeed, extruded granules containing CN NS were characterized by a faster dissolution rate with respect to the reference samples containing CN powder as such (Figure 5c), even in a poorly discriminating media for the drug of interest, such as the acidic buffer pH 1.2 (i.e., a media in which drug solubility is relatively high). Also in this case, the CN amorphous quote was associated with dissolution of the drug in the polymer. In this respect, to increase the amount of crystalline matter within the product, it would be necessary to extrude the formulation at temperatures largely below drug T_m_. However, this was hard to achieve due to the high polymer melt viscosity at lower temperatures with respect to those employed.

#### 3.3.3. PEO 303

**Figure 6:**
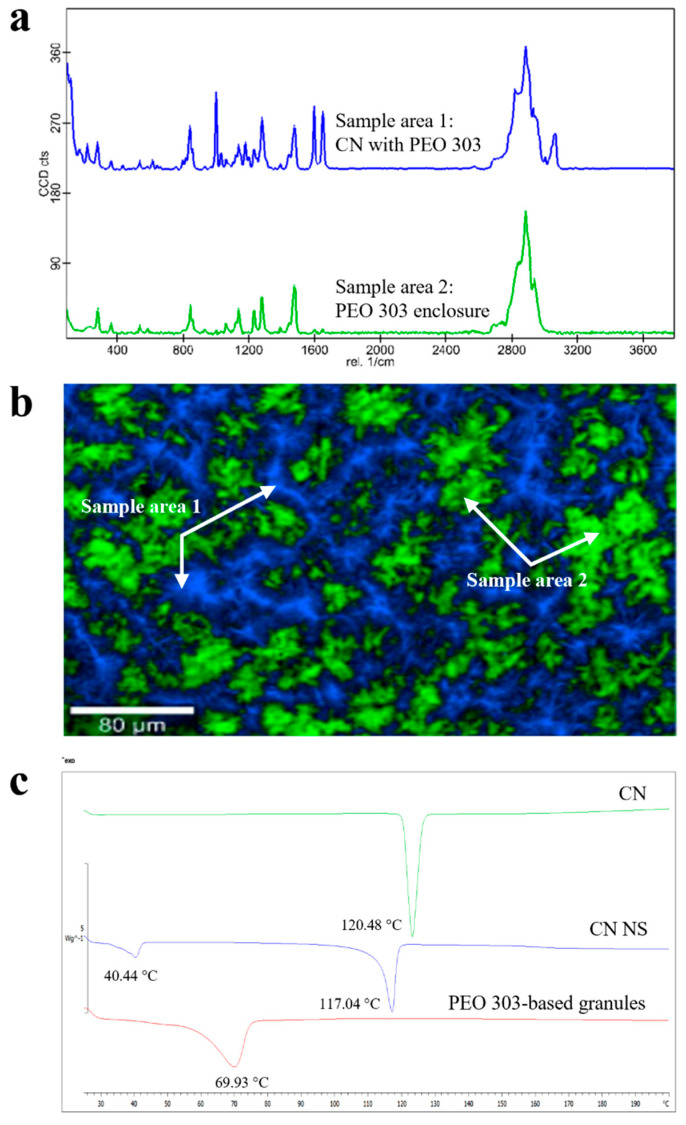
(**a**) Raman molecular map, (**b**) Raman spectra and (**c**) DSC thermograms of PEO 303-based granules.

A high molecular weight PEO (i.e., PEO 303), which is widely employed in the formulation of swellable/erodible PR matrices, was also screened. Focusing on Raman data, areas in which CN was embedded within the PEO matrix (i.e., blue sample area 1 in Figure 6a,b) could be highlighted, the latter surrounding well-demarcated and segregated enclosures with a predominant polymeric composition (i.e., green sample area 2 in Figure 6a,b). Although the presence of shoulders of diagnostic peaks at the lattice vibration frequencies in the CN spectra may alert to the presence of undefined quantities of crystalline CN, the absence of the endothermic peak corresponding to CN melting in DSC profiles indicated its quantitative amorphization (Figure 6c). Working temperatures for this high molecular weight PEO turned out especially critical. In fact, the 100 °C set during HME failed in ensuring complete melting of the polymer, but caused CN dissolution.

#### 3.3.4. Eu RS, Eu RL and Eu E

High-resolution Raman analysis of Eu-based extruded products pointed out a spatial distribution of pure crystalline CN entities into the different polymethacrylate-based carriers. More specifically, there were three diverse scenarios.

**Figure 7:**
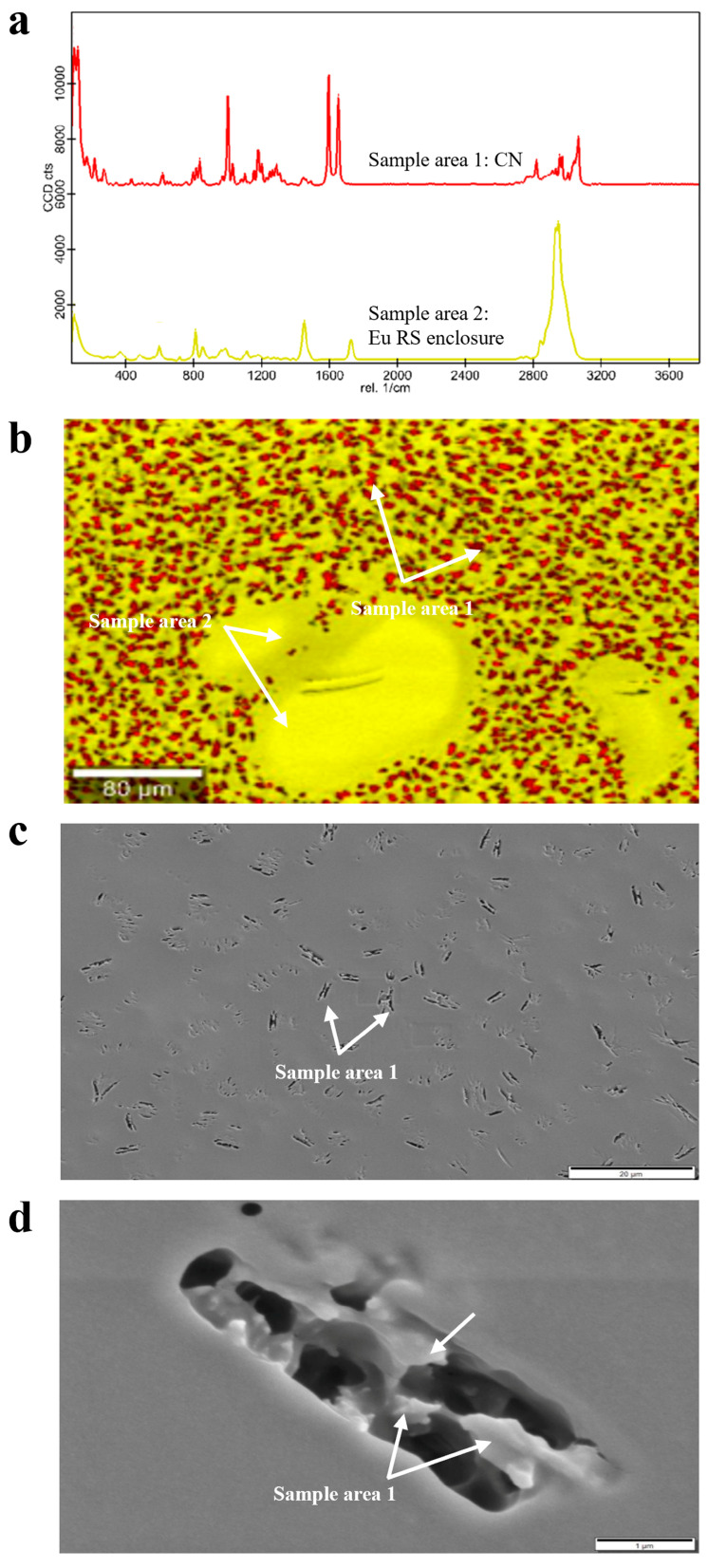
(**a**) Raman spectra, (**b**) Raman molecular map and (**c**,**d**) SEM images, at different magnifications, of Eu RS-based granules.

In Eu-RS-based granules, finely and randomly dispersed regions of pure CN were observed, which were immersed in the pure polymeric carrier (i.e., red and yellow sample areas 1 and 2, respectively, in Figure 7a,b). To better clarify the solid state of CN spots within the polymer, SEM images were also acquired. Interestingly, the images of the sample cross-section showed elongated pore clusters with a butterfly-like pattern, in the walls of which CN crystals could be appreciated via appropriate magnification (Figure 7d). Individually, these crystalline entities measured up to 0.5–1.0 μm. On the other hand, the presence of the pores could be associated with rapid drying of residues of the liquid fraction of the CN NS. These large pores being randomly dispersed within the matrix could be ascribed to CN NS fractions whose aqueous part was not properly mixed with the polymer and was dried too fast during HME (Figure 7c).

**Figure 8:**
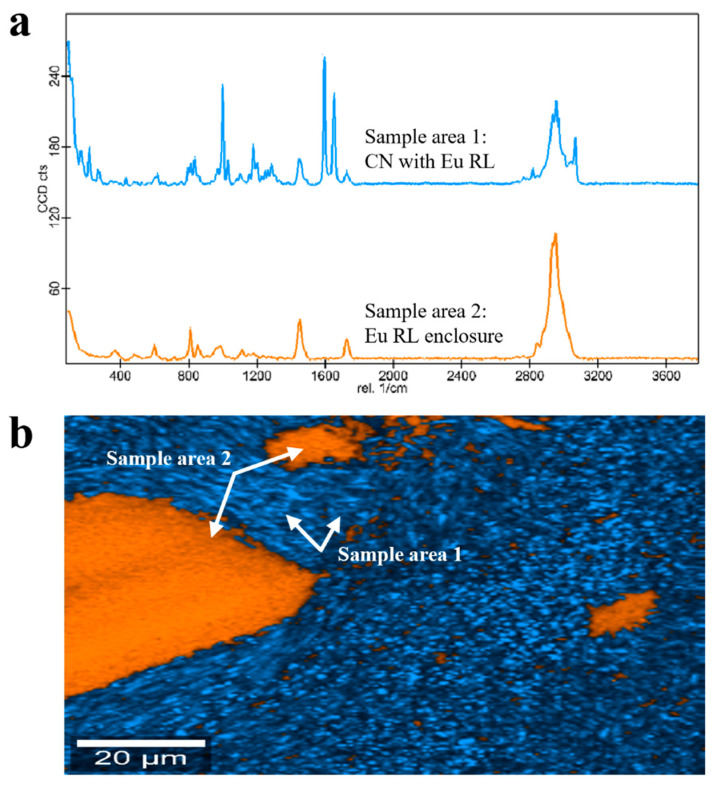
(**a**) Raman spectra and (**b**) Raman molecular map of Eu RL-based granules.

**Figure 9:**
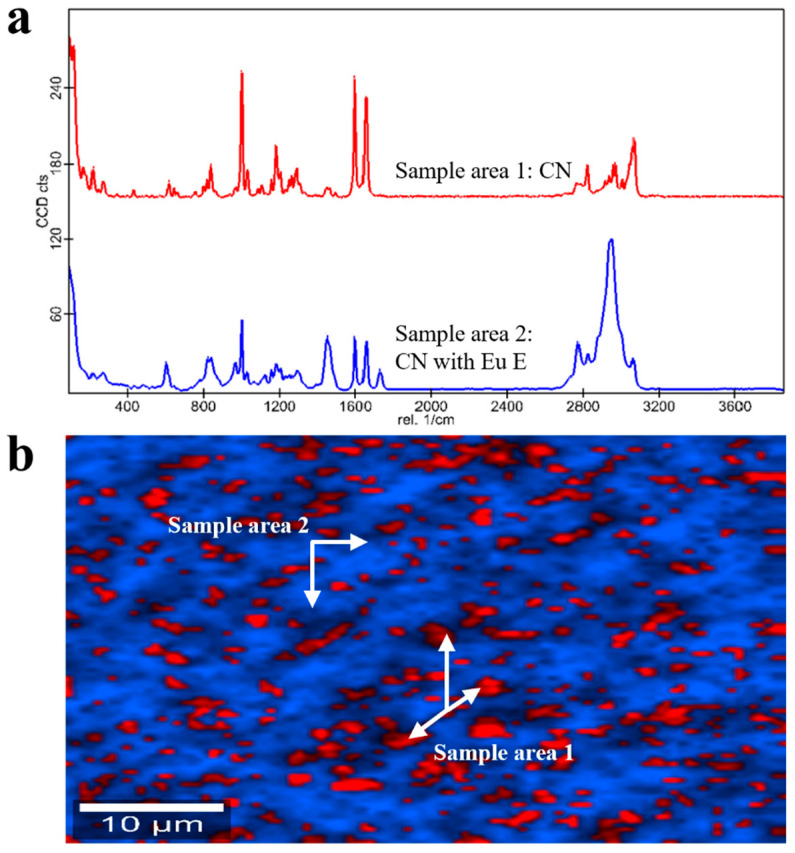
(**a**) Raman spectra and (**b**) Raman molecular map of Eu E-based granules.

In the case of Eu RL-based granules, Raman findings revealed two main molecular entities: a crystal CN dispersion in the polymer (i.e., blue sample area 1 in Figure 8a,b) with Eu RL enclosures having a length of ~1–340 μm (i.e., orange sample area 2 in Figure 8a,b). As previously discussed for PEG- and PEO 303-based units, pure unaltered polymeric regions in polymethacrylate-based samples could be ascribed to the low residence time of the formulations in the lab-scale extruder employed, which did not allow a complete phase transition at the selected temperature and plasticizing conditions. This was not the case of Eu E-based products. In fact, the Raman analysis pointed out an intimate dispersion of CN in the polymer (i.e., blue sample area 1 in Figure 9a,b) but with enclosures of pure crystalline drug (i.e., red sample area 2 in Figure 9a,b).

## 4. Conclusions

This work represented a proof-of-concept study on the feasibility of HME in manufacturing multi-particulate systems for oral intake having a variety of release behavior, starting from a NS based on CN, a BCS class II model drug. In a wider perspective, this work further confirmed the versatility of HME for both small-scale manufacturing, as required by clinical trials testing the potential of NCEs, and for large-scale processing. Indeed, it was demonstrated a useful technology to turn NSs into solid products in a single step, thus being compatible with continuous manufacturing, while enabling the attainment of granules with both IR and PR performances. Compared with what was already available in the scientific literature, this research contributed to widen the numerosity and type of polymeric carriers that can be employed for processing NSs via HME. As CN represented a quite demanding model drug in view of its thermal behavior, it was challenging to attain granules containing the unmodified nanocrystals and equally interesting to identify the conditions under which CN dissolved and amorphized into the carrier. In this respect, the combination of the different analytical techniques used in the work was also proven fundamental in deepening the understanding of the products’ micro-structure.

## Figures and Tables

**Table 1:** HME parameters relevant to different formulations.

Formulation		HME Conditions			Granulation-Oven Drying Steps, n°
Polymeric Carrier	Plasticizer, % on the Dry Carrier	T, °C	Screw Speed, rpm	Max Torque Recorded, N·cm	
EXP	10% H_2_O and 15% GLY	90	35	150	1
PEO N10	-	80	30	15	5
PEO 303	-	100	15	90	2
PEG	-	50–55	15	85	6
Eu RL	10% TEC	80	15	90	2
Eu RS	15% TEC	85	30	15	2
Eu E	5% TEC	80	30	30	2
EMD	10% GLY	50	30	40	6

## Data Availability

The original contributions presented in this study are included in the article. Further inquiries can be directed to the corresponding author.

## Abbreviations

Biopharmaceutical classification systems	BCS
Cinnarizine	CN
Cinnarizine nanosuspension	CN NS
Coefficient of variation	CV
Confocal Raman microscopy	CRM
D-a-tocopheryl polyethylene glycol 1000 succinate	TPGS
Differential scanning calorimetry	DSC
Dynamic light scattering	DLS
Drug delivery system	DDS
Eudragit^®^	Eu
Emdex^®^	EMD
Explotab^®^ CLV	EXP
Fourier-transform infrared	FT-IR
Gastrointestinal	GI
Glass transition temperature	T_g_
Glycerol	GLY
High-performance liquid chromatography	HPLC
Hot melt extrusion	HME
Immediate release	IR
Melting temperature	T_m_
New chemical entity	NCE
Nanosuspension	NS
Polydispersity index	PdI
Polyethylene oxide	PEO
Polyethylene glycol	PEG
Prolonged release	PR
Scanning electron microscopy	SEM
Triethyl citrate	TEC
Wet media milling	WMM
